# A Cortical Folding Pattern-Guided Model of Intrinsic Functional Brain Networks in Emotion Processing

**DOI:** 10.3389/fnins.2018.00575

**Published:** 2018-08-21

**Authors:** Xi Jiang, Lin Zhao, Huan Liu, Lei Guo, Keith M. Kendrick, Tianming Liu

**Affiliations:** ^1^The Clinical Hospital of Chengdu Brain Science Institute, MOE Key Lab for Neuroinformation, School of Life Science and Technology, University of Electronic Science and Technology of China, Chengdu, China; ^2^School of Automation, Northwestern Polytechnical University, Xi'an, China; ^3^Department of Computer Science and Bioimaging Research Center, University of Georgia, Athens, GA, United States

**Keywords:** emotion, task fMRI, intrinsic functional network, cortical gyri and sulci, functional model

## Abstract

There have been increasing studies demonstrating that emotion processing in humans is realized by the interaction within or among the large-scale intrinsic functional brain networks. Identifying those meaningful intrinsic functional networks based on task-based functional magnetic resonance imaging (task fMRI) with specific emotional stimuli and responses, and exploring the underlying functional working mechanisms of interregional neural communication within the intrinsic functional networks are thus of great importance to understand the neural basis of emotion processing. In this paper, we propose a novel cortical folding pattern-guided model of intrinsic networks in emotion processing: gyri serve as global functional connection centers that perform interregional neural communication among distinct regions via long distance dense axonal fibers, and sulci serve as local functional units that directly communicate with neighboring gyri via short distance fibers and indirectly communicate with other distinct regions via the neighboring gyri. We test the proposed model by adopting a computational framework of dictionary learning and sparse representation of emotion task fMRI data of 68 subjects in the publicly released Human Connectome Project. The proposed model provides novel insights of functional mechanisms in emotion processing.

## Introduction

Understanding the neurobiological basis of emotions (e.g., fear, anger, sadness, etc.) in humans has received extensive interests in the affective neuroscience field (Lindquist and Barrett, 2012; Lindquist et al., 2012). With the advancement of *in-vivo* functional neuroimaging techniques such as functional magnetic resonance imaging (fMRI) (Logothetis, 2008; Friston, 2009) as well as the development of advanced image analysis and computational modeling methodologies, researchers are able to examine the neural circuitry of emotion processing for a better understanding of the functional architecture of brain emotion. Specifically, based on task fMRI with specific emotional stimuli and responses, specific brain regions or brain networks involved in such emotion processing can be identified; in other words, it is assumed that different kinds of emotion processing can be localized to specific brain regions/networks (Vytal and Hamann, 2010; Panksepp, 2011; Lindquist et al., 2012; Murphy et al., 2012). Recently, mounting evidence has shown that human brain is intrinsically organized into multiple functional networks such as default mode, visual, motor, auditory, cognitive control, etc., each of which is spatially distributed across specific neuroanatomical areas (Fox et al., 2005; Bullmore and Sporns, 2009; Duncan, 2010; Pessoa, 2012; Fedorenko et al., 2013); the emotion processing is realized by the interaction within or among those intrinsic functional brain networks (Bressler and Menon, 2010; Lindquist and Barrett, 2012; Barrett and Satpute, 2013). As a consequence, identifying meaningful intrinsic functional brain networks based on task fMRI data, as well as exploring its underlying functional working mechanisms of interregional neural communication, is of great importance to understand the neural basis of emotion processing.

**Figure 1:**
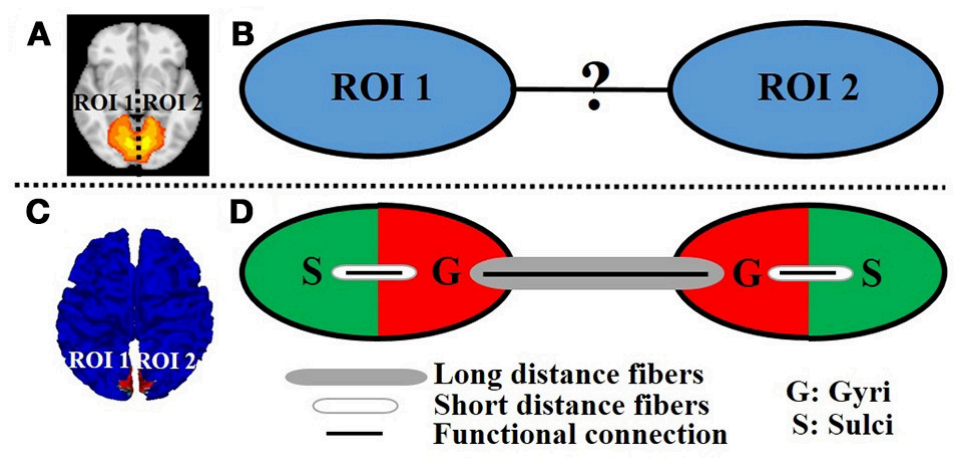
An example intrinsic network composed of two distinct regions of interest (ROI 1 and 2 in left and right hemisphere, respectively) viewed in volume space **(A)** and on cortical surface **(C)**. **(B)** The unknown functional working mechanisms of interregional neural communication within one network. **(D)** Illustration of the proposed model. The red/green color represents the gyral/sulcal regions within the ROI of intrinsic network.

A variety of fMRI time series analysis methodologies have been successfully applied in the brain mapping field for intrinsic functional network identification based on either task fMRI data or resting state fMRI data such as principal component analysis (PCA) (Andersen et al., 1999), independent component analysis (ICA) (McKeown et al., 1998), and dictionary learning/sparse representation (Abolghasemi et al., 2015; Lv et al., 2015a,b). The premise is that the activity patterns in fMRI blood-oxygen level-dependent (BOLD) signals among spatially distinct brain regions within an intrinsic network are temporally coupled. Figure 1A shows an example intrinsic network which is composed of two spatially distinct brain regions (regions of interest (ROI) 1 and 2 in left and right hemisphere, respectively). Although the distinct regions within one network are argued to be functionally linked and interacting with each other, the underlying functional working mechanisms of interregional neural communication among those regions within one network are still largely unknown (Figure 1B).

In the literature of brain network analysis, however, there has been little effort devoted to adding the factor of cortical folding patterns into consideration. Actually, the cortical folding pattern, which is composed of highly convoluted convex gyri and concave sulci, is one of the most prominent features of human brain (Barron, 1950; Welker, 1990). A variety of studies have demonstrated that there are both structural and functional differences between cortical gyral and sulcal regions (Nie et al., 2012; Chen et al., 2013; Deng et al., 2014; Zhang et al., 2014; Jiang et al., 2015, 2018). For example, it is reported that gyral regions are connected by much denser diffusion tensor imaging (DTI) or high angular resolution diffusion imaging (HARDI) derived axonal fiber bundles than sulcal regions in the whole cortex, indicating that gyri are structural connection center of the cortex (Nie et al., 2012; Chen et al., 2013; Zhang et al., 2014). Another studies report that gyral regions have stronger functional connectivity and more spatial overlap patterns of global functional networks than sulcal regions, indicating that gyri are global functional center of the cortex (Deng et al., 2014; Jiang et al., 2015, 2018).

As an attempt to modeling the interregional neural communication of intrinsic networks in emotion processing, and inspired by the abovementioned structural/functional differences between gyral and sulcal regions as well as the previous finding that distinct regions within one intrinsic network are interconnected by DTI-derived fiber bundles (Greicius et al., 2009; Van den Heuvel et al., 2009), in this paper, we propose a novel cortical folding pattern-guided model of the intrinsic network (Figure 1C) in emotion processing: gyri serve as global functional connection centers that perform interregional neural communication among distinct regions via long distance dense fibers, and sulci serve as local functional units that directly communicate with neighboring gyri via short distance fibers (inter-column cortico-cortical fibers) and indirectly communicate with other distinct regions via the neighboring gyri with the dense fibers (Figure 1D). We test the proposed model by assessing the task fMRI signal representation accuracy via a computational framework of dictionary learning and sparse representation of whole-brain emotion task fMRI signals. We hypothesize that the sparse representation accuracy value of task fMRI signals, which indicates the degree of interregional neural communication among distinct regions, is significantly larger on gyri than on sulci within the intrinsic network in emotion processing.

## Materials and methods

### Data acquisition and pre-processing

We adopt the emotion task fMRI data of 68 subjects in the publicly released Human Connectome Project (HCP, Q1 release) (Barch et al., 2013) as a testbed in this paper. This emotion task is similar with the one in Hariri et al. (2002). Participants were presented and asked to match either two different shapes or faces (with angry or fearful expressions) at the bottom of the screen with the one at the top of the screen. There were six blocks (of face or shape alternatively), each of which was preceded by a 3 s task cue (shape or face) and 6 trials of the same match task (face or shape, 3 s for each trial). There were 3 face blocks and 3 shape blocks for each of the two runs. More details of the task design are referred to Barch et al. (2013).

The acquisition parameters of the task fMRI data are as follows (Barch et al., 2013): 220 mm FOV, 90 × 104 matrix, 72 slices, TR = 0.72 s, 176 volumes (time points), TE = 33.1 ms, flip angle = 52, in-plane FOV = 208 × 180 mm, 2.0 mm isotropic voxels. The pre-processing steps using FSL FEAT are referred to Barch et al. (2013) which mainly include skull removal, motion correction, slice time correction, spatial smoothing, and global drift removal (high-pass filtering).

### Sparse representation of whole-brain task fMRI signals

We adopt a computational framework of dictionary learning and sparse representation of whole-brain task fMRI signals to test the proposed model. The rationales are 2-fold. First, the dictionary learning and sparse representation framework has been demonstrated as an efficient and effective data-driven approach in identifying concurrent intrinsic networks based on task fMRI signals (Abolghasemi et al., 2015; Lv et al., 2015a,b) (detailed in Section Intrinsic network identification and representation on cortical surface). This is also the premise to model the working mechanisms of intrinsic networks. Second, the dictionary learning and sparse representation framework can learn meaningful functional activity basis patterns from hundreds of thousands of whole-brain task fMRI signals effectively and represent the task fMRI signals based on the learned basis efficiently and compactly (Abolghasemi et al., 2015; Lv et al., 2015a,b). Assessing the task fMRI signals representation accuracy based on the learned basis in gyral/sulcal regions within one intrinsic network is reasonable to validate the proposed cortical folding pattern-guided model of intrinsic network in emotion processing as detailed in Section Signal representation accuracy assessment on gyri/sulci within one intrinsic network.

**Figure 2:**
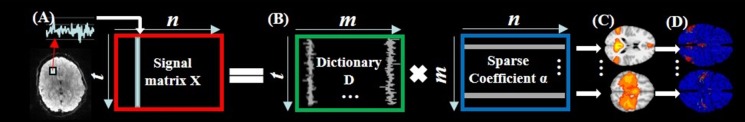
The illustration of sparse representation of whole-brain rsfMRI signals. **(A)** The whole-brain rsfMRI signals of an example subject which are aggregated into a 2D matrix **X**. **(B)** The decomposed dictionary matrix **D** and sparse coefficient matrix **α** based on **X**. **(C)** The identified intrinsic networks in task fMRI volume space. **(D)** The corresponding intrinsic networks on cortical surface.

As illustrated in Figure 2, for each subject, first, the fMRI signals of whole-brain voxels are extracted, and normalized to zero mean and standard deviation of 1 (Mairal et al., 2010). Second, all *n* normalized signals, each of which has *t* time points, are aggregated into a 2D matrix **X** = [**x**_1_, **x**_2_,…,**x**_*n*_]ϵℝ^*t* × *n*^ (Figure 2A). Third, by applying the widely adopted online dictionary learning method (Mairal et al., 2010), **X** is decomposed into an over-complete dictionary matrix **D** = [**d**_1_, **d**_2_,…,**d**_*m*_]ϵℝ^*t* × *m*^ (*m* is the dictionary size, *m* > *t* and *m* < < *n*) and a sparse coefficient matrix α = [α_1_, α _2_,…, α _*n*_]ϵℝ^*m* × *n*^ (Figure 2B). In this way, each fMRI signal is represented as a linear combination of all learned dictionary atoms in **D**, i.e., **x**_*i*_ = **D** × α_*i*_ + ε (ε is error term). Specifically, an empirical cost function *f*_*n*_(**D**) of **X** is defined to assess the average loss of regression of all *n* signals based on **D**:

(1) $$\begin{array}{r} f_{n}\left(\text{D}\right)≜\frac{1}{n}\overset{n}{\underset{i=1}{\sum }}l\left({\text{x}}_{i},\text{D}\right) \end{array}$$

where $l\left({\text{x}}_{i},\text{D}\right)≜\underset{\alpha_{i}\epsilon {\mathbb{R}}^{m}}{\min}\frac{1}{2}{\left||{\text{x}}_{i}-\text{D}\alpha_{i}|\right|}_{2}^{2}+\lambda {\left||\alpha_{i}|\right|}_{1}\;$. Note that the *l*_1_ regularization is adopted for a sparse solution of **α**_*i*_. λ is used to regularize regression loss and sparsity level. We also defined a constraint for **D** to make the coefficients in α comparable:

(2) $$\begin{array}{r} C≜\left\{\text{D}\epsilon {\mathbb{R}}^{t\times m}\;s.t.\;i=1,\ldots m,\;{\left({\text{d}}_{i}\right)}^{T}{\text{d}}_{i}\leq 1\right\} \end{array}$$

In this way, Equation (1) can be rewritten as a matrix factorization problem:

(3) $$\begin{array}{r} \underset{\text{D}\epsilon C,\alpha \epsilon {\mathbb{R}}^{m\times n}}{\min}\frac{1}{2}{\left||\text{X}-\text{D}\alpha |\right|}_{F}^{2}+\lambda {\left||\alpha |\right|}_{1,1\;} \end{array}$$

We learn **D** in Equation (3) using the effective online dictionary learning method (Mairal et al., 2010). α is then solved based on **D** as an *l*_1_ regularized linear least-squares problem (Mairal et al., 2010). We use the parameter setting of the same HCP data in Lv et al. (2015b) as *m* = 400 and λ = 1.5. From brain science perspective, the learned dictionary atoms in **D** represent a set of signal basis (Figure 2B) derived from whole-brain task fMRI signals. Each original fMRI signal can be represented by these relevant signal basis patterns via linear combination.

### Intrinsic network identification and representation on cortical surface

As demonstrated in Section Sparse representation of whole-brain task fMRI signals, each column of α stores the sparse coefficients of representing each original fMRI signal based on **D**. Moreover, each row of α can be mapped back to the original brain volume space to represent the spatial volumetric pattern that has reference to each dictionary atom (Figure 2C). To identify the meaningful intrinsic networks from all spatial patterns, we adopt the publicly available intrinsic network template (Smith et al., 2009) as references. This template provides nine stable and meaningful intrinsic networks on cortical area including three visual, default mode, motor, auditory, executive control, and bilateral frontal/parietal networks (Smith et al., 2009). Specifically, the spatial pattern similarity is defined as the overlap rate *R*:

(4) $$\begin{array}{r} R\left(S,T\right)=\;|S\cap T|/|T| \end{array}$$

where *S* is a set of cortical vertices involving in a spatial pattern that has reference to a dictionary atom, *T* is a set of cortical vertices involving in the spatial pattern of a specific intrinsic network template (Smith et al., 2009). The spatial pattern with the highest *R* with the intrinsic network template is identified as the corresponding intrinsic network in this individual subject as previous studies (Lv et al., 2015b). Note that the task-induced network can also be effectively identified and separated with intrinsic networks by means of considering both temporal and spatial patterns of dictionary atoms in the dictionary learning and sparse representation framework as detailed in Jiang et al. (2015, 2018) and Lv et al. (2015a,b). We then map the identified intrinsic networks in task fMRI volume space (Figure 2C) to T1 cortical surface (Figure 2D) in order to utilize the cortical folding pattern information. Specifically, the network is firstly converted into T1 volume space and then mapped onto the cortical surface using an in-house tool by localizing each voxel involved in the network to its nearest cortical mesh vertex.

### Signal representation accuracy assessment on gyri/sulci within one intrinsic network

As demonstrated in Section Sparse representation of whole-brain task fMRI signals, the learned over-complete **D** represents a set of all basis components of neural activities from whole-brain task fMRI signals. Each original fMRI signal **x**_*i*_ is approximately represented as ${\bar{\text{x}}}_{i}=$ **D** × α_*i*_. Here we assess the task fMRI signal representation accuracy *P* as:

(5) $$\begin{array}{r} P_{{\text{x}}_{i}}=corr\left({\bar{\text{x}}}_{i},{\text{x}}_{i}\right) \end{array}$$

where corr(.) is the Pearson's correlation coefficient between **x**_*i*_ and **x**_i_ and ranges from 0 to 1. The larger the *P* is, the better the signal representation is for **x**_*i*_, i.e., **x**_*i*_ can be well represented by the basis components of neural activities in **D**. In other words, **x**_*i*_ well participates in or follow the neural activities in emotion processing. Since the distinct regions within one intrinsic network theoretically have similar neural activities and are functionally linked, the assessment of the task fMRI signal representation accuracy (Equation 5) in these distinct regions within the intrinsic network is thus indicative of the degree of interregional neural communication among distinct regions within the intrinsic network.

Specifically, for each distinct region *V* = ∀*v*_*i*_ (*v*_*i*_ is the cortical vertex in the region) within one intrinsic network on cortical surface, we first calculate the signal representation accuracy value *P*_*v*_*i*__ (Equation 5) for the task fMRI signals of all *v*_*i*_. Second, based on the principal curvature value of *v*_*i*_ to delineate gyral/sulcal regions as $pcurv_{v_{i}}\{\begin{array}{c} \geq 0,\;v_{i}\epsilon gyri\\ <0,v_{i}\epsilon sulci \end{array}\;$ provided in the HCP data (Barch et al., 2013), we separate *V* into gyral and sulcal regions as *V*_*gyri*_ = ∀*v*_*i*_ *s*.*t*. *pcurv*_*v*_*i*__ ≥ 0 and *V*_*sulci*_ = ∀*v*_*i*_ *s*.*t*. *pcurv*_*v*_*i*__ < 0, respectively. Note that *V* = *V*_*gyri*_ + *V*_*sulci*_. Finally, the set of all signal representation accuracy values in gyral and sulcal regions is represented as *P*_*V*_*gyri*__ = ∀*P*_*v*_*i*__ *s*.*t*. *v*_*i*_ ∈ *V*_*gyri*_ and *P*_*V*_*sulci*__ = ∀*P*_*v*_*i*__ *s*.*t*. *v*_*i*_ ∈ *V*_*sulci*_, respectively. By evaluating the possible mean accuracy value difference between gyral and sulcal regions in each of the distinct regions within one intrinsic network, the proposed cortical folding pattern-guided model of the intrinsic network in emotion processing is validated.

## Results

### Signal representation accuracy difference on gyri/sulci in default mode network

**Figure 3:**
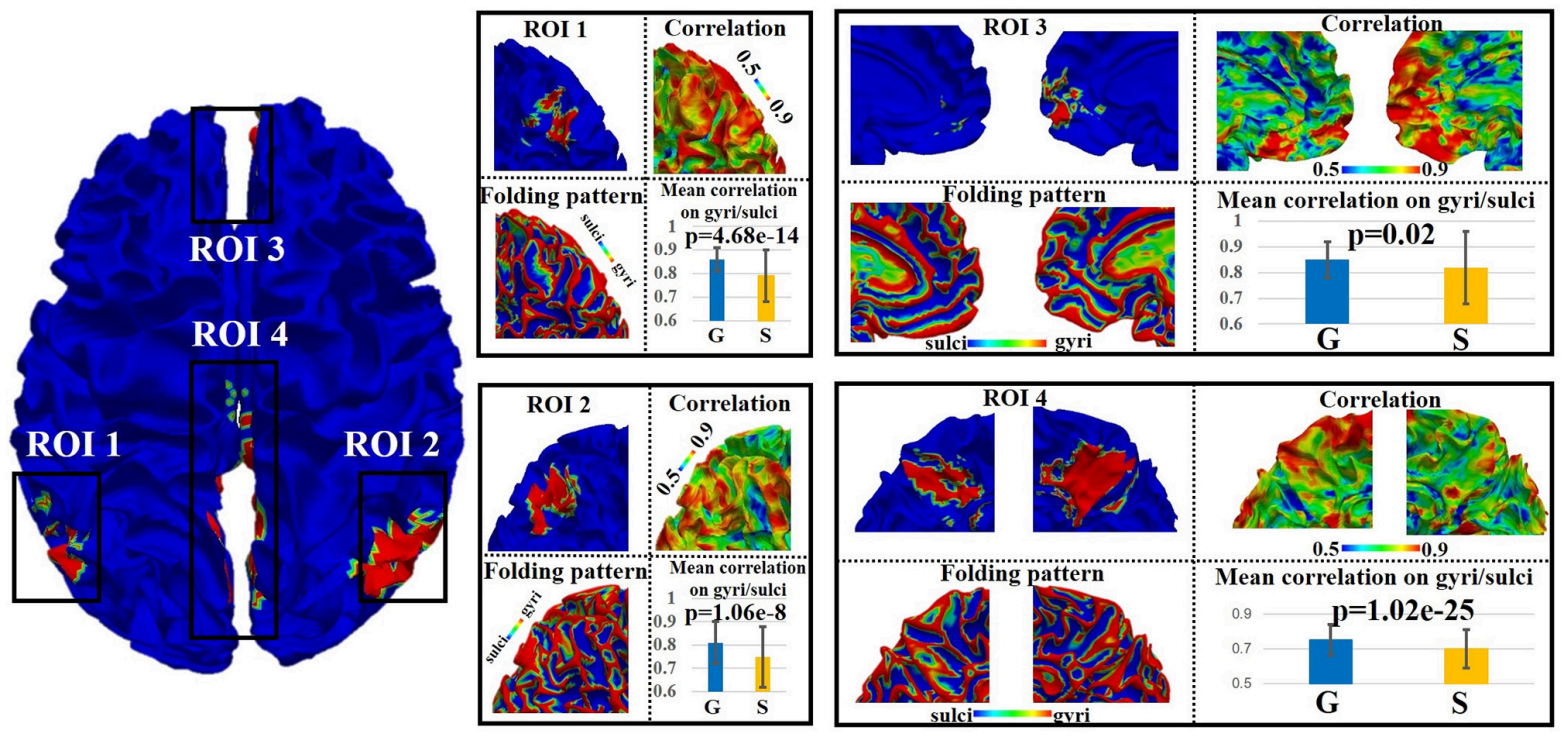
Emotion task fMRI signal representation accuracy difference between gyral and sulcal regions in default mode network (DMN) of one subject. The detailed assessment of each of the four distinct regions (ROI 1–4) within DMN is in zoomed-in view. G, gyri; S, sulci. *P*-value: two-sample one-tailed *t*-test (gyri > sulci, *p* = 0.05, Bonferroni corrected).

We adopted the proposed framework to examine the signal representation accuracy difference on gyri/sulci in default mode network (DMN), which is one of the most recognized intrinsic network (Smith et al., 2009). As illustrated in Figure 3, there are four spatially distinct regions of interest (ROIs) in DMN including left inferior parietal lobule (ROI 1), right inferior parietal lobule (ROI 2), bilateral medial prefrontal gyrus/anterior cingulate cortex (ROI 3), and bilateral posterior cingulate cortex (ROI 4) (Smith et al., 2009). For each of the four ROIs, we can see that both gyral and sulcal regions have reasonably high accuracy value (the mean accuracy value is 0.82 for gyri and 0.76 for sulci) since the sparse representation approach (Section Sparse representation of whole-brain task fMRI signals) can relatively effectively represent whole-brain rsfMRI signals. However, there is still accuracy difference between gyral and sulcal regions. A two-sample one-tailed *t*-test between the set of accuracy values of gyri and sulci (*p* < 0.05, Bonferroni corrected) shows that the signal representation accuracy value on gyri is significantly larger than that on sulci for each of the four ROIs (Figure 3).

### Signal representation accuracy difference on gyri/sulci in other intrinsic networks

**Figure 4:**
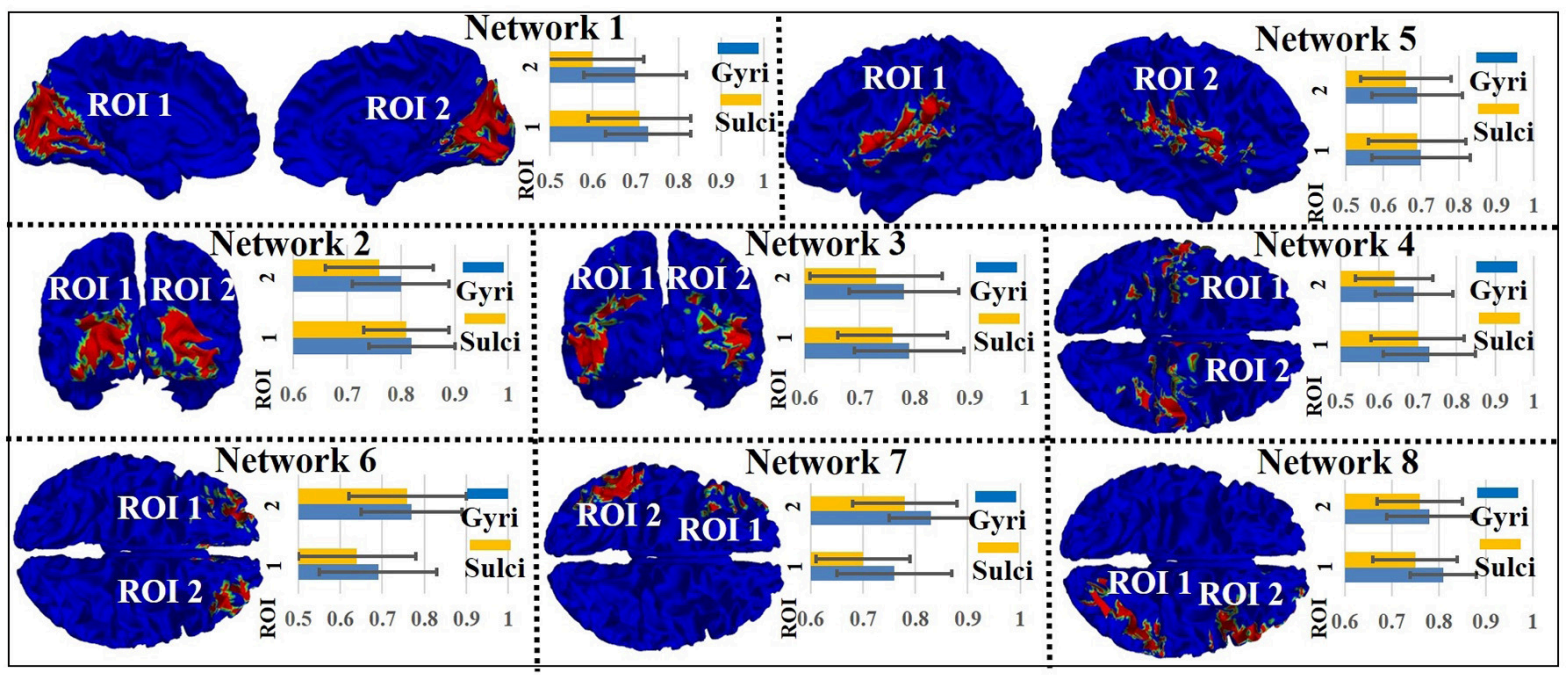
Emotion task fMRI signal representation accuracy difference between gyral and sulcal regions in the other eight intrinsic networks.

We assessed the signal representation accuracy on gyri/sulci in the other eight intrinsic networks to examine the generality of the proposed working model of intrinsic networks in emotion processing. As shown in Figure 4, the eight intrinsic networks (Smith et al., 2009) include three visual networks (Network 1–3), motor (Network 4), auditory (Network 5), executive control (Network 6), and bilateral frontal/parietal networks (Network 7–8). The mean accuracy value across all ROIs in all eight intrinsic networks is 0.75 for gyri and 0.71 for sulci. A two-sample one-tailed *t*-test between the set of accuracy values of gyri and sulci (*p* < 0.05, Bonferroni corrected) shows that the signal representation accuracy value on gyri is significantly larger than that on sulci in each of the intrinsic network.

### Reproducibility and structural substrates of the intrinsic functional network model

We assessed the signal representation accuracy on gyri/sulci in all nine intrinsic networks in emotion processing in all 68 subjects. Table 1 indicates that the signal representation accuracy on gyri is consistently larger than sulci in each of the nine intrinsic networks across a majority of subjects. Moreover, we performed the permutation test for each intrinsic network to separate all signal representation accuracy values within the intrinsic network into gyri and sulci groups and to calculate the mean difference between the two groups for 1000 times. The *p*-value based on the 1,000-time permutation *t*-test is *p* < 0.05 for all intrinsic networks and subjects, indicating the signal representation accuracy of gyri is truly larger than sulci within all intrinsic networks in emotion processing.

**Table 1:** Proportion of number of subjects with significant gyral/sulcal signal representation accuracy difference (two-sample *t*-test, *p* < 0.05, Bonferroni corrected) in the intrinsic networks in emotion processing.

Network	1	2	3	4	5	6	7	8	9
Proportion	0.67	0.82	0.76	0.78	0.75	0.99	0.82	0.82	0.97

*Network 1–3, three visual networks; Network 4, motor; Network 5, auditory; Network 6, executive control; Network 7–8, bilateral frontal/parietal networks; Network 9, default mode*.

We further adopted the DTI data of the same 68 subjects in the HCP data set to examine the correlation between DTI FA value and the signal representation accuracy in gyri/sulci. The experimental results show that across all intrinsic networks and subjects, the FA values are positively correlated with the emotion task signal representation accuracy in gyri/sulci (*r*-value ranges from 0.3 to 0.6 across different intrinsic networks and subjects, *p*-value < 0.01), indicating that gyri has both more structural fiber connections and higher task fMRI signal representation accuracy than sulci.

## Discussion

We proposed a novel cortical folding pattern-guided model of intrinsic functional brain networks in emotion processing. This model is evaluated and validated via the proposed computational framework of dictionary learning and sparse representation of emotion task fMRI signals, and the task fMRI signal representation accuracy assessment on gyral and sulcal regions within the intrinsic network. Experimental results based on the HCP emotion task fMRI data demonstrated that the fMRI signal representation accuracy value in gyri is significantly larger than that on sulci across all nine major cortical intrinsic networks. Our results provide novel insights of functional mechanisms in emotion processing.

We identified nine meaningful intrinsic functional networks which mainly locate on cortical regions based on the emotion task fMRI data. Note that we focus on the intrinsic networks on cortical regions in this work in order to take advantage of the cortical folding pattern information. There are also meaningful and important intrinsic networks in subcortical area (e.g., amygdala, etc.) in emotion processing for future studies. Our finding is consistent with previous studies showing that there are “*domain-general, distributed*” intrinsic functional networks in human brain, and emotion processing arises from the interaction within or among these intrinsic functional brain networks (Lindquist and Barrett, 2012; Barrett and Satpute, 2013).

We found that the emotion task fMRI signal representation accuracy value is significantly larger on gyral regions than sulcal regions within the intrinsic network, indicating that gyri might directly participate more than sulci in functional activities/interactions among distinct regions within the intrinsic networks in emotion processing. As demonstrated in Section Signal representation accuracy assessment on gyri/sulci within one intrinsic network, the learned dictionary matrix represents a set of all basis neural activities of emotion task fMRI signals in the whole-brain. The gyral regions with higher signal representation accuracy based on all basis neural activities are thus of more neural communication among distinct regions within the intrinsic network, i.e., gyral regions serve as global neural communication centers among distinct regions within the intrinsic network in emotion processing. The sulcal regions with lower signal representation accuracy are thus of less neural communication among distinct regions within the intrinsic network and serve as local centers within the single regions of the intrinsic network in emotion processing. This finding, to some extent, is in agreement with the previous studies arguing that gyral regions have stronger interregional functional connectivity than sulcal regions (Deng et al., 2014). It is also in agreement with other studies demonstrating that gyral regions have more spatial overlap patterns of functional networks than sulcal regions in both temporally stationary and dynamic states (Jiang et al., 2015, 2018). Moreover, we found that the DTI FA values are positively correlated with the emotion task signal representation accuracy in gyri/sulci. Given the fact that brain structure predicts its function (Passingham et al., 2002; Zhang et al., 2011), this finding as well as other mounting evidences that gyral regions have more interregional axonal fiber connections than sulcal regions (Nie et al., 2012; Chen et al., 2013; Zhang et al., 2014) provide the structural substrates for the abovementioned functional observations. In conclusion, based on both structural and functional evidences, we argue that the emotion task fMRI signal representation accuracy difference between gyri and sulci within the intrinsic network reasonably supports our proposed cortical folding pattern-guided model; that is, within an intrinsic network in emotion processing, gyri are the global functional connection centers which perform interregional neural communication among distinct regions, and sulci are the local functional units which directly communicate with neighboring gyri and indirectly communicate with other distinct regions via the neighboring gyri.

In this work, we adopted the emotion task fMRI data in the HCP datasets as a testbed. The proposed model showed reproducibility and generality across different subjects under the same emotion task design. In the future, we plan to test our model on other task fMRI data sets with different emotion processing paradigms to examine if there is any common finding among different emotion processing paradigms. It would be also interesting to explore the potential general principle of our proposed cortical folding pattern-guided model using different task data sets.

## Author contributions

XJ designed the experiment, analyzed part of the data, generated figures, and wrote the manuscript. LZ analyzed part of the data. HL pre-processed and analyzed part of the data. LG guided HL and LZ on this work. KK interpreted the results and revised the manuscript. TL designed the experiment and critically revised the manuscript.

### Conflict of interest statement

The authors declare that the research was conducted in the absence of any commercial or financial relationships that could be construed as a potential conflict of interest.
